# Roles Played by DOCK11, a Guanine Nucleotide Exchange Factor, in HBV Entry and Persistence in Hepatocytes

**DOI:** 10.3390/v16050745

**Published:** 2024-05-08

**Authors:** Ying-Yi Li, Kazuhisa Murai, Junyan Lyu, Masao Honda

**Affiliations:** 1 Department of Gastroenterology, Kanazawa University Graduate School of Medicine, 13-1, Takaramachi, Kanazawa 920-8640, Japan; 2 Department of Clinical Laboratory Medicine, Kanazawa University Graduate School of Health Medicine, 13-1, Takaramachi, Kanazawa 920-8640, Japan

**Keywords:** DOCK11, hepatitis B virus, retrograde trafficking, cccDNA persistence

## Abstract

HBV infection is challenging to cure due to the persistence of viral covalently closed circular viral DNA (cccDNA). The dedicator of cytokinesis 11 (DOCK11) is recognized as a guanine nucleotide exchange factor (GEF) for CDC42 that has been reported to be required for HBV persistence. DOCK11 is expressed in both the cytoplasm and nucleus of human hepatocytes and is functionally associated with retrograde trafficking proteins Arf-GAP with GTPase domain, ankyrin repeat, and pleckstrin homology domain-containing protein 2 (AGAP2), and ADP-ribosylation factor 1 (ARF1), together with the HBV capsid, in the trans-Golgi network (TGN). This opens an alternative retrograde trafficking route for HBV from early endosomes (EEs) to the TGN and then to the endoplasmic reticulum (ER), thereby avoiding lysosomal degradation. DOCK11 also facilitates the association of cccDNA with H3K4me3 and RNA Pol II for activating cccDNA transcription. In addition, DOCK11 plays a crucial role in the host DNA repair system, being essential for cccDNA synthesis. This function can be inhibited by 10M-D42AN, a novel DOCK11-binding peptide, leading to the suppression of HBV replication both in vitro and in vivo. Treatment with a combination of 10M-D42AN and entecavir may represent a promising therapeutic strategy for patients with chronic hepatitis B (CHB). Consequently, DOCK11 may be seen as a potential candidate molecule in the development of molecularly targeted drugs against CHB.

## 1. Introduction

DOCK11 (dedicator of cytokinesis 11), also referred to as Zizimin2 (Ziz2), was originally discovered as a guanine nucleotide exchange factor (GEF) predominantly expressed in mouse lymphoid tissues [1]. In 2006, it was reported that a ~220 kDa protein (p220) formed a stable complex with activated forms of CDC42 (cell division cycle 42) in the mouse embryonic teratoma cell line P19; this was designated as activated CDC42-associated GEF (ACG). Sequence alignment against the NBCI GenBank database revealed that p220 (ACG) corresponded to DOCK11, a member of the DOCK180 family of GEFs [2]. The human DOCK11 (NCBI accession number NP_653259.3) shares 96% identity and 98% similarity with the murine protein and does not exhibit any splice isoforms.

DOCK11 is one of the three members of the DOCK-D family, along with DOCK9 (Zizimin1) and DOCK10 (Zizimin3) [1,2,3,4]. The first member of the DOCK-D family, DOCK9, was initially named Zizimin1, a term derived from the Hebrew word “zizim”, meaning spikes, due to its ability to induce microspikes in fibroblasts through direct interaction with CDC42 [3]. Subsequently, DOCK11 and DOCK10 were successively identified through cloning efforts [1,2,4]. DOCK11 shares significant sequence homology with human DOCK9 (58%) and DOCK10 (50%) at the amino acid level, including within kinase domains.

**Figure 1:**
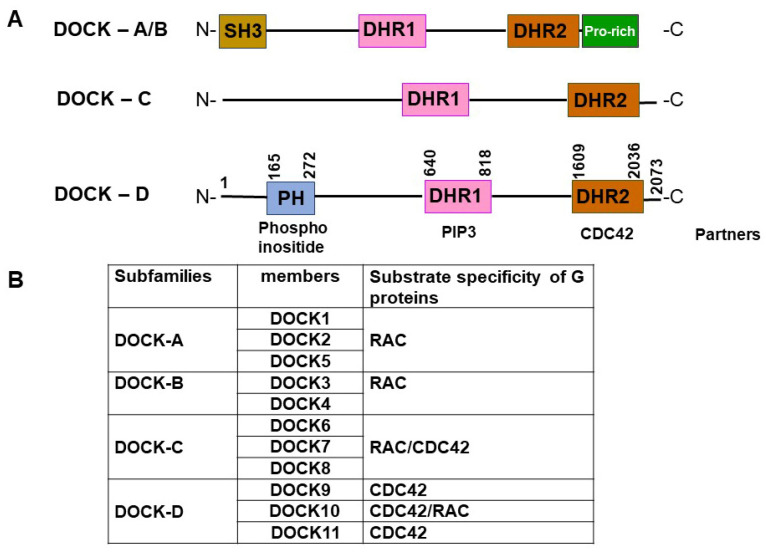
**Schematic of DOCK subfamilies, their domain architecture structure, and substrate specificity.** (**A**) The DOCK GEF family is subdivided into four subfamilies. All DOCK proteins contain a DHR1, mediating binding to PIP3, and the DHR2 domain, encompassing GEF activity. Members of the DOCK-A and DOCK-B subfamilies possess an SH3 domain at the N-terminus and a proline-rich (Pro-rich) region at the C-terminus. Members of the DOCK-C and DOCK-D subfamilies contain neither the SH3 domain nor the Pro-rich region, but DOCK-D subfamily proteins possess a PH domain at the N-terminus. (**B**) DOCK proteins are categorized into four subgroups according to their evolutionary relationships and substrate specificity.

To date, 11 members of the DOCK protein family have been identified; these have been classified into four subgroups [5] based on sequence similarity, associated regulatory domains, and substrate specificity (Figure 1). The DOCK-A subgroup consists of DOCK1 (also known as DOCK180), DOCK2, and DOCK5; the DOCK-B subgroup includes DOCK3 (also known as PBP (presenilin binding partner) or MOCA (modifier of cell adhesion)) and DOCK4. Although the DOCK-A/B subgroups share similar structures, characterized by an amino-terminal SH3 domain capable of binding ELMO (engulfment and motility) adaptor proteins and a proline-rich carboxy-terminal domain that binds Crk proteins [6], their classification into DOCK-A and -B subfamilies was determined on the basis of their identity scores; these were obtained through ClustalW and phylogenetic analysis conducted using their sequences [7]. The DOCK-C subgroup comprises DOCK6, DOCK7, and DOCK8, also known as Zir (for Zizimin-related) 1, 2, and 3, respectively. Finally, the DOCK-D subgroup consists of DOCK9, DOCK10, and DOCK11, also known as Zizimin (Ziz) 1, 3, and 2, respectively, all of which possess an amino-terminal PH domain [8]. DOCK-A/B proteins act as exchange factors for the GTPase RAC, while DOCK-C proteins are considered dual-specificity GEFs for RAC and CDC42 [9]. Among the DOCK-D proteins, DOCK9 and DOCK11 exhibit a preference for interacting with CDC42, whereas DOCK10 interacts with both RAC and CDC42 [10,11].

DOCK proteins, functioning as guanine nucleotide exchange factors, play a crucial role in regulating small GTPases that serve as pivotal mediators in numerous cellular processes [5,8,12,13]. Extensive research efforts have underscored the potential of DOCK proteins as therapeutic targets in various fields, such as cancer, immunology, and neurology [14,15,16]. However, to date, there have been no published reports in the literature directly linking members of the DOCK family with viral infections. Patients with DOCK8 deficiency often experience cutaneous viral infections involving such viruses as varicella zoster, molluscum contagiosum, herpes simplex, and human papilloma. In such cases, DOCK8 deficiency may affect both innate and adaptive immune responses [17]. In light of this, in the present review, we primarily focus on a single DOCK-D family member, DOCK11, and consider its significant cellular functions, particularly its involvement in hepatitis B virus (HBV) entry, trafficking, and covalently closed circular viral DNA (cccDNA) synthesis within hepatocytes.

## 2. Structure of the DOCK11 Protein

The open reading frame of DOCK11 mRNA encodes a protein consisting of 2073 amino acids with a calculated molecular weight of 237,671 Da [1]. The DHR1 domain of DOCK11, which spans approximately 200 residues and is positioned near the N terminus, exhibits a C2-like architecture and plays a pivotal role in binding to PtdIns(3,4,5)P3 to facilitate the recruitment of DOCK complexes to the cell membrane, particularly at leading cellular edges where signaling events initiate motility [18]. The catalytic DHR2 domain, which spans approximately 500 residues and is located at the C-terminal region, serves as the primary site for GEF activity (Figure 1A). The N-terminal PH domain of DOCK11 has been reported to interact with phosphoinositides of the plasma membrane (PM) [19]. In addition, recent findings suggest that in DOCK-D proteins, the PH domain may function analogously to the PH domain of ELMO1 (ELMO protein 1), a cofactor of the DOCK-A and DOCK-B subfamilies. Specifically, it may interact with GTPase bound to the DHR2 domain, potentially enhancing GEF activity and/or contributing to GTPase specificity, as predicted by ALPHFOLD2 [11]. However, in DOCK11, the binding-activated forms of CDC42 are located within amino acid residues 66–126 of the N-terminal region. The PH domain does not directly mediate this binding, nor does it confer high GEF activity [2]. Interestingly, in previous studies, co-localization and co-immunoprecipitation of DOCK11 with AGAP2 have been detected, suggesting the potential formation of a complex between DOCK11 and AGAP2, given that AGAP2 possesses a PH domain similar to the ELMO1 protein [6,20]. However, the precise role played by the PH domain within the DOCK-D family is yet to be fully articulated.

GTPases cycle between an active state, bound to guanosine triphosphate (GTP) under the influence of GEFs, and an inactive state, bound to guanosine diphosphate (GDP) under the influence of GTPase-activating proteins (GAPs) [21]. The full-length crystal structure of the DOCK-D subfamilies has not yet been obtained. However, the crystal structure of the DHR2 domain in DOCK9 in complex with CDC42 was reported by the authors of [22]. This structure revealed that the conserved DHR2 domain is organized into three distinct lobes, named A, B, and C. Lobe A consists of helical repeats, mediates the homodimerization of DOCK, and stabilizes lobe B. Lobes B and C serve as binding sites for CDC42. Specifically, residue Vall1951 within Helix α10 of lobe C acts as a nucleotide sensor, mediating the release of GDP and, subsequently, the discharge of activated (GTP-Mg^2+^) bound CDC42 from the DHR2 [22]. DOCK recognizes its substrates via lobe C, which serves as the active center of the GEF reaction, and lobe B, which recognizes switch 1 of the small GTPase. In previous research, we discovered that ARF1, a member of the ARF family, can form a complex with DOCK11 and AGAP2. DOCK11 interacts with ARF1 via switch 1 and exhibits GEF activity toward ARF1. AGAP2 interacts with ARF1 proteins and exhibits high affinity and GAP activity toward ARF1 [23]. This binding of DOCK11 and AGAP2 might be beneficial for recruiting ARF1 to DOCK11 and AGAP2 and thus promoting cycling between GTP-bound ARF1 and GDP-bound ARF1 [20].

## 3. Subcellular Localization and Expression Regulation of DOCK11

The DOCK11 protein has been detected in various human tissues, including the brain, gastrointestinal tract, liver, gallbladder, bone marrow, and lymphoid tissues (as reported by The Human Protein Atlas). The cellular localization of endogenous DOCK11 protein has been observed in the cytosol and nucleoplasm of lymphocytes, myeloid cells, and 293T cells. The balance between the two compartments varies and is specific to the cell line [24]. This variability suggests that the subcellular localization of DOCK11 could facilitate interactions with distinct partners and give rise to diverse potential functions. Specifically, in previous works, we found that DOCK11 is expressed in both the nucleus and cytoplasm in hepatocarcinoma cells and primary human hepatocyte (PXB) cells under HBV infection conditions [20,25]. The authors of [26] found that, in HepAD38 cells in which HBV expression was regulated by the CMV-tet promoter, DOCK11 exhibited clear perinuclear localization adjacent to lamin A/C, indicating its involvement in HBV retrograde trafficking in the cytoplasm and maintenance of cccDNA in the nucleoplasm.

In mouse lymphoid tissue, both the Fcγ (Fc gamma) receptor and TLR4 (toll-like receptor 4) have been shown to upregulate and stabilize DOCK11 expression, thereby promoting filopodial formation via CDC42 activation in bone marrow-derived dendritic cells, indicating that DOCK11 plays a role in immune system modulation [27]. In addition, because the expression of DOCK11 is regulated by age-dependent mechanisms, any age-associated downregulation of DOCK11 in B cells could impact secondary immune responses [28]. Furthermore, DOCK11 mRNA and protein expressions exhibit good correspondence [29], suggesting that regulation of DOCK11 expression could be relatively straightforward. Interestingly, we observed a significant increase in the expression of DOCK11 at both mRNA and protein levels in HBV-infected cells compared with non-infected cells [20]. We also found that DOCK11 levels in liver biopsies from patients with chronic hepatitis B were significantly reduced by entecavir (ETV) treatment, and this reduction correlated with HBV surface antigen levels [20]. Additionally, analysis of a publicly available dataset on human liver tissue (GEO: GSE83148) revealed that the expression of DOCK11 in hepatocytes was influenced by HBV infection and was increased in patients with chronic hepatitis B [26], possibly suggesting potential involvement in HBV replication and cccDNA maintenance.

## 4. Roles of DOCK11 in HBV Cell Entry and Persistence in Hepatocytes

Chronic hepatitis B affects over 300 million people worldwide. It is a major cause of liver disease, resulting in approximately 800,000 deaths each year [30]. HBV infection is challenging to cure due to the persistence of cccDNA. The HBV life cycle can be generally divided into two phases: (1) an early phase that includes attachment/entry, trafficking to the nucleus, and cccDNA formation; and (2) a late replication phase consisting of transcription, encapsulation, reverse transcription, envelopment, and release [31]. cccDNA acts as HBV’s reservoir, serving as the sole template for viral RNAs, notably pgRNA, which is then transcribed into rcDNA, forming the viral genome [32,33]. Given cccDNA’s pivotal role in HBV replication, identifying early-stage molecular targets and developing targeted therapies could potentially achieve a full or partial cure of chronic hepatitis B (CHB). We performed single-cell transcriptome analysis of newly established HBV-positive and HBV-negative hepatocellular carcinoma cell lines and found that DOCK11 was crucially involved in HBV persistence [34].

### 4.1. DOCK11 Enhances Early Stages of HBV Life Cycle

**Figure 2:**
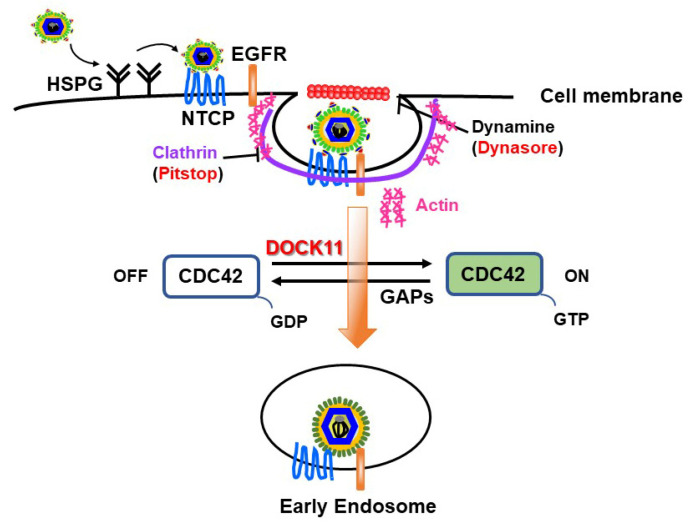
**The proposed hypothesis explains the mechanism for HBV entry into early endosomes.** DOCK11 might exert its effect by modulating CDC42 GTPase activity, thereby inducing actin polymerization and subsequently promoting HBV endocytosis on the cell surface [20].

We previously observed that ablation of DOCK11 expression by shDOCK11 in PXB cells significantly suppressed HBV DNA and cccDNA levels [20]. Furthermore, when we used recombinant NanoLuc (NL)-HBV particles, which mimic the early stage of the HBV life cycle from entry to transcription/translation through cccDNA formation [35], we observed that the substantial increase in cccDNA-derived Luc activity resulting from DOCK11 overexpression was markedly repressed by the entry inhibitors Dynasore (an endocytosis inhibitor) and Pitstop 2 (a clathrin inhibitor). For HBV entry into host hepatocytes, it may be necessary for HBV particles to attach to susceptible cells through heparan sulfate proteoglycans (HSPGs) factors [36] and then bind to its receptors, sodium taurocholate cotransporting peptides (NTCPs), thus mediating HBV endocytosis [37]. The mechanism for endocytosis of epidermal growth factor receptor (EGFR) coordinates the transport of incoming hepatitis B virus to the endosomal network [38]. This step depends on cellular factors such as clathrin, caveolin-1, and dynamin-2. Notably, the actin cytoskeleton is essential for the formation and movement of clathrin-associated endocytic vesicles [39,40,41]. Thus, DOCK11 likely exerts its effects by modulating CDC42 GTPase activity, thereby inducing actin polymerization and subsequently promoting HBV endocytosis on the cell surface (Figure 2) [2].

### 4.2. DOCK11 and AGAP2 Regulate ARF1 to Promote the Retrograde Trafficking of HBV in the Cytoplasm

Following endocytosis, HBV viral particles travel from early endosomes to late endosomes/lysosomes associated with the cellular RAB GTPases RAB5A and RAB7A, where virus uncoating occurs [42]. We previously confirmed that the HBV core-related antigen (HBcAg) is positively localized in RAB7-positive structures [20]. To examine the functional relevance of RAB7 for HBV infection, we established the HepG2-NTCPC4-Rab7 knockout (RAB7KO) cell line. Surprisingly, we observed that the loss of RAB7 actually increased intracellular levels of HBV DNA and cccDNA after HBV infection, which were abrogated by Retro-2 treatment, a retrograde trafficking inhibitor. We also found that a lysosome inhibitor (chloroquine) did not decrease HBV infection but rather increased its replication [20]. In light of these findings, we hypothesized that another trafficking pathway was used by HBV to reach the nucleus and initiate replication by escaping HBV endosomal-lysosomal degradation.

**Figure 3:**
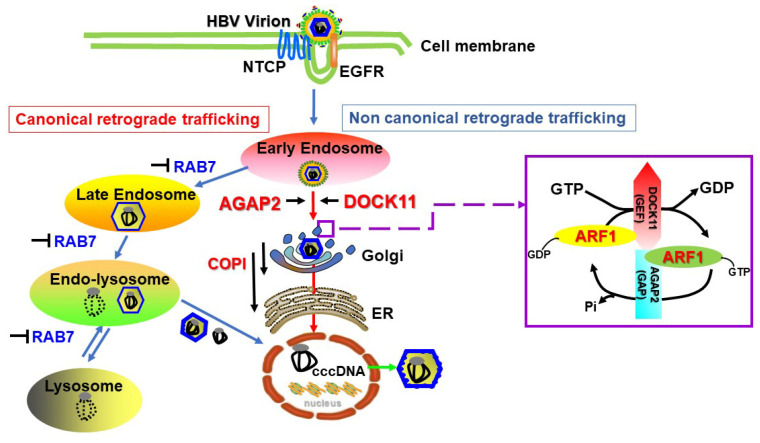
**Schematic representation for DOCK11 and its partner AGAP2 regulating ARF1 activity to facilitate HBV retrograde trafficking**. HBV typically enters cells via the canonical retrograde trafficking route, involving initial binding to the NTCP receptor, followed by encapsulation of the virus-receptor complex within early endosomes. Subsequently, the complex is transported to late endosomes and lysosomes, where viral particles are exposed to lysosomal enzymes. Notably, capsid proteins, including the viral genome, evade lysosomal degradation and may enter the nucleus [42]. Additionally, we have uncovered a non-canonical retrograde trafficking route from early endosomes to the trans-Golgi network and endoplasmic reticulum, which facilitates HBV entry into the nucleus. Importantly, DOCK11 and its partner AGAP2 are implicated in regulating ARF1 activity to facilitate this HBV retrograde trafficking pathway [20].

The retrograde transport from endosomes to the trans-Golgi network serves to divert proteins and lipids away from lysosomal degradation; these include Shiga toxin, cholera toxin, and human papillomavirus 16 [43]. For more information on the retrograde trafficking of proteins and pathogens, readers are referred to pertinent reports [44,45]. Furthermore, we revealed that DOCK11 regulates AGAP2, facilitating HBV retrograde transport from the EE-TGN-endoplasmic reticulum (ER) pathway to the nucleus for viral genome replication [20]. ARF1 was initially reported to regulate intracellular vesicular trafficking from the Golgi apparatus to the ER and to cycle between an active GTP-bound form and an inactive GDP-bound form during cargo trafficking [46]. We observed that the DOCK11-DHR2 domain exhibited GEF activity and that AGAP2 exhibited GAP activity toward ARF1 [20]. Thus, DOCK11 and AGAP2 modulate ARF1 activity to facilitate the retrograde trafficking of HBV from the EE-TGN-ER pathway, thereby bypassing the canonical trafficking route involving lysosomal degradation and subsequent entry into the cell nucleus to sustain HBV replication (Figure 3).

Currently, the means by which virion-derived nucleocapsids that have escaped from late endosomes are delivered to the nucleus are understood through two models. One model involves lysosome and HBV envelope membrane fusion, leading to the release of the nucleocapsids into the cytoplasm [47]; the other involves a translocation process mediated by the cell-permeable peptide at the PreS2 domain, designated as the translocation motif (TLM) [48,49]. The release of nucleocapsids to the nucleus might also be affected via microtubule-mediated transport [48]. There is also a lack of information regarding how the nucleocapsid released from the ER enters the nucleus. Connections between the ER and the nuclear membrane have been identified, and one recent report showed that lipid droplets (LDs) generated in the ER lumen can move into the nucleoplasm of hepatocytes through a precise molecular mechanism [50]. It has also been reported that HBV capsids combined with LDs can efficiently establish infection [51]. Moreover, ARF1/COPI machinery acts directly on LDs and enables their connection to the ER [52]. In previous work, we discovered that DOCK11 and AGAP2 could form a complex, and the depletion of COPI prevented HBV capsid trafficking via the TGN-ER pathway [20]. Therefore, ER stress-induced lipid droplets in the ER could potentially participate in the retrograde trafficking of HBV via the EE-TGN-ER pathway to the cell nucleus. Further analyses should enable us to provide a more detailed molecular mechanism of HBV nuclear entry regulated by DOCK11.

### 4.3. Predicted Roles of DOCK11 in DNA Repair and HBV cccDNA Formation in the Nucleus

After delivery into the nucleus and uncoating, the relaxed circular DNA (rcDNA) is expected to prompt recognition of its structural anomalies at the termini as DNA damage signals by the host DNA repair machinery, thereby facilitating conversion into cccDNA, which serves as a template for viral transcription and pregenomic RNA synthesis by host RNA polymerase II [33,53]. cccDNA formation involves several steps. First, the rcDNA is recognized by the ataxia telangiectasia and the Rad3-related (ATR) pathway, one of the cellular DNA damage response pathways. Removal of covalently bound polymerase by tyrosyl-DNA phosphodiesterase 2 (TDP2) [54] and the RNA primer is then followed by DNA synthesis through host DNA polymerase (Polκ, Polσ, and Polα). Subsequently, after cleavage of the redundant region “r” region at (-) strand DNA by flap structure-specific endonuclease 1 (FEN1), the ligation forms closed cccDNA by DNA ligases 1 and 3 (LIG 1 and LIG 3) [33]. In addition, proliferating cell nuclear antigen (PCNA) and replication factor C (RFC) are also involved in lagging strand synthesis and DNA repair [55].

**Figure 4:**
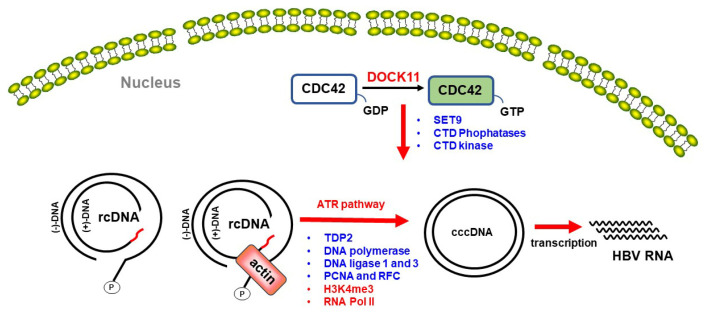
**The proposed hypothesis explains the mechanism for the role of DOCK11 in HBV cccDNA formation and its modulation of histone modifications and RNA Pol II on HBV cccDNA**. DOCK11 activates CDC42 to stimulate actin polymerization, thereby activating the ATR signaling pathway and facilitating cccDNA formation [25]. Additionally, DOCK11 triggers the CDC42-mediated pathway, regulating downstream molecules such as SET9 (a lysine methyltransferase), C-terminal domain (CTD) phosphatases, and CTD kinase to modulate H3K4 methylation and phosphorylation status within the RNA Pol IIA CTD, ultimately maintaining cccDNA [26].

In studies by Ide et al., Doan et al., and ourselves, using super-resolution microscopy analysis, it was shown that DOCK11 is expressed not only in the cytoplasm but also in the nucleus [20,25,26]. Previous studies have also shown that actin filament accumulation at sites of DNA damage is essential for ATR assembly [56]. Ide et al. also showed that DOCK11 participates in actin polymerization in both the cytoplasm and the nucleus and that DOCK11 colocalizes with γH2AX, a marker for DNA damage sites, following UV irradiation in the nucleus of HepG2 cells [25]. This might suggest that DOCK11 accumulates at DNA damage sites in response to DNA damage signals and promotes actin polymerization, thereby activating the ATR signaling pathway in the cell nucleus. In HBV-infected cells, DOCK11 may activate the ATR signaling pathway in this manner, thus facilitating cccDNA formation (Figure 4, left) [25].

HBV cccDNA is a stable episomal form of the HBV viral genome decorated with host histone and non-histone proteins. Histone modifications on cccDNA play a pivotal role in regulating viral transcription [57,58]. Methylation of histone H3 lysine 4 (H3K4) typically correlates with gene activation, whereas methylation of H3K27 is commonly associated with gene silencing due to its role in chromatin condensation. Nucleosomal HP1α serves as a transcriptional repressor, in contrast with RNA Pol II, which facilitates active transcription [57,58]. Using an in situ hybridization method [59], Doan et al. revealed that DOCK11 increased HBV DNA and cccDNA levels in HepAD38 and HepG2.2.15 cells. However, although DOCK11 did not directly colocalize with H3K4me3, H3K27me3, RNA Pol II CTD (C-terminal domain), or HP1α using super-resolution microscopy analysis, ChIP-qPCR results demonstrated that shDOCK11 led to a significant reduction in the enrichment of H3K4me3 and RNA Pol II CTD on HBV cccDNA, while HP1α and H3K27me3 remained unaffected [26]. The precise mechanism by which DOCK11 modulates the interaction of H3K4me3 and RNA Pol II CTD with HBV cccDNA remains elusive. A previous investigation suggested that RHO-family GTPases, including CDC42, have the capacity to modify the phosphorylation status of Ser2 and Ser5 residues within the CTD of RNA Pol IIA, consequently impacting gene expression (Figure 4, right) [60]. Hence, it has been proposed that DOCK11 activates the RHO GTPase CDC42-mediated pathway. Nevertheless, a more comprehensive understanding of this mechanism is imperative.

## 5. Biological Functions of DOCK11 in Other Pathological Conditions

Transforming growth factor beta 1 (TGF-β1) is an important growth inhibitor for epithelial cells. TGF-β1 also promotes the growth of some fibroblasts and is currently understood to be the principal mediator of the fibrotic response in the liver. TGF-β1 signals can activate the Smad pathway via the phosphorylation of Smad2 and Smad3. The authors of [61] reported that DOCK9 was co-immunoprecipitated with Smad2 and Smad3 in response to TGF-β1. Additionally, the expression of AGAP2 has been shown to modulate some of the pro-fibrotic effects described for TGF-β1 in the liver [23]. In light of the formation of a complex between DOCK11 and AGAP2 and the regulatory role played by DOCK11 on AGAP2 expression, it is conceivable that DOCK11, along with AGAP2, may serve as a novel participant in the signaling pathway of the pro-fibrotic cytokine TGF-β1. This suggests a potential contribution of DOCK11 to the advancement of hepatic fibrosis progression.

While metastatic cancer cells develop the capacity to migrate and disseminate from the primary tumor, individual cancer cells can employ either a mesenchymal or an amoeboid mode of migration [62]. In melanoma cells, DOCK10 promotes amoeboid migration by promoting the activation of CDC42 and its downstream effectors, WASl (also known as N-Wasp) and PAK2 [13]. The closely related DOCK11 and DOCK10, belonging to the same subfamily, exhibit 66% identity and 83% similarity in the DHR2 domain, suggesting their potential involvement in promoting cancer cell migration and infiltration through the activation of CDC42 or other GTPases. Indeed, abnormal expression of DOCK11 has been found in testicular carcinoma in situ [63]. Because DOCK11 has been shown to recruit TLR4, leading to the activation of CDC42 and the promotion of cell migration, and TLR4 has been implicated in promoting epithelial-mesenchymal transition (EMT) and the migration of cancer cells, we may speculate that DOCK11 is associated with cancer-induced pathological cell migration.

In recent studies, Block et al. and Boussard et al. were the first to report that DOCK11 deficiency in humans caused a new X-linked immune-related actinopathy, which, in the human DOCK11 gene, is located on chromosome X [64,65]. Across the two studies, a total of 12 patients presented with early-onset autoimmunity and autoinflammation, including skin inflammation, severe gastrointestinal disease, cytopenia, and systemic lupus erythematosus. DOCK11 is known to be involved in leukocyte diapedesis and interstitial migration [64]. Such homing and migration alterations in T-cell subsets could contribute to infection susceptibility and severity [64]. Future research efforts should shed light on the molecular mechanisms of DOCK11-underlying inflammatory diseases, including HBV infection.

## 6. Pharmacologic Inhibition of DOCK11

The targeting of GEFs by chemical compounds is an important approach used for the discovery of drugs that block RHO or ARF GTPases. An increasing body of evidence suggests that the DOCK family, including DOCK 11, plays a pivotal role in regulating diverse fundamental biological processes and is implicated in various diseases, ranging from auto-immune disease and HBV infection to neurodegeneration. This underscores the need for the discovery of chemical inhibitors that could serve as therapeutic interventions.

Recent studies have identified three chemical leads that block the activation of RAC1 by members of the DOCK-A subfamily, including C21 [66,67], CPYPP [68], and TBOPP [69]. Recently, Ide et al. employed the in vitro virus (IVV) method to discover novel DOCK11-binding peptides and antibodies against the hepatocyte-specific asialoglycoprotein receptor (ASGR). Using this approach, they identified the DOCK11-binding peptide DCS8-42A, which exhibits anti-HBV activity, and also the anti-ASGR antibody ASGR3-10M, which demonstrates a high affinity for ASGR, making it suitable for hepatocyte-specific uptake [25]. Ide et al. also designed a fusion protein named 10M-D42AN, comprised of scFv ASGR3-10M, a Furin cleavage sequence, the DOCK11-binding peptide DCS8-42A, the fusogenic peptide S28, and a nuclear localization signal (NLS) [25]. It was speculated that upon endocytosis in the hepatocyte mediated by the anti-ASGR antibody, the construct would be routed to early endosomes, where it would undergo cleavage by Furin, leading to dissociation of the peptide. Subsequently, it was anticipated that the peptide would be released into the cytoplasm via the fusogenic peptide, thereby inhibiting the interaction between DOCK11 and its partner proteins in both the cytoplasm and nucleus. Moreover, 10M-D42AN was found to inhibit HBV internalization by suppressing EGFR endocytosis on the cell surface, consistent with the effect of the entry inhibitors [20], and to suppress HBV replication in PXB cells [25].

In addition, it was found that 10M-D42AN administered with entecavir reduced HBV DNA and cccDNA levels compared with the administration of entecavir alone in vitro. Further evidence for this effect is supplied by our observation that knocking down DOCK11 in combination with ETV treatment further suppressed HBV replication, compared with either factor alone, in HepAD38 and HepG2.2.15 cells [20]. In conclusion, the hepatocyte-specific delivery of the DOCK11 inhibitor may contribute to the development of safe and usable drugs for suppressing HBV infection.

## 7. Future Perspectives

The broadening of our understanding of GTPase pathways beyond the well-studied RHO family to include other members like ARF and their interactions with DOCK GEFs is crucial for advancing our comprehension of cellular signaling networks. Increased knowledge in these areas could contribute to the development of novel therapeutic strategies for a wide range of diseases, including those related to viral infections like HBV.

In addition, more in-depth research into the physiological roles played by DOCK11 within the immune system offers the possibility of enhancing immune responses against infections, including HBV. By elucidating the intricate signaling pathways mediated by DOCK11, targets may be identified for therapeutic interventions aimed at bolstering immune defense mechanisms.

It is worth noting that DOCK proteins, including DOCK11, have emerged as potential therapeutic targets for various diseases in addition to viral infections. Conditions such as glaucoma, Alzheimer’s disease, cancer, attention deficit hyperactivity disorder (ADHD), and combined immunodeficiency are just some of the areas where targeting DOCK proteins has shown promise. Further research into the roles and regulatory mechanisms of DOCK proteins could lead to the development of novel therapeutic approaches for these diverse disorders.
